# Risk factors associated with renal injury in patients initially diagnosed with IgA vasculitis

**DOI:** 10.3389/fped.2025.1584768

**Published:** 2025-07-29

**Authors:** Lu Shen, Li Miao, Lian Xu

**Affiliations:** Department of Pediatrics, the First People’s Hospital of Lianyungang, The Affiliated Lianyungang Hospital of Xuzhou Medical University, The First Affiliated Hospital of Kangda College of Nanjing Medical University, Lianyungang Clinical College of Nanjing Medical University, Lianyungang, Jiangsu, China

**Keywords:** child, immunoglobulin a vasculitis (IgAV), immunoglobulin a vasculitis nephritis (IgAVN), total cholesterol (TC), albumin (ALB), risk factor, prediction model

## Abstract

**Objective:**

To explore the risk factors associated with renal injury in patients diagnosed with IgA vasculitis at initial presentation.

**Methods:**

A retrospective analysis was conducted on the clinical data of 384 children who were newly diagnosed with Immunoglobulin A vasculitis (IgAV) and hospitalized between July 2020 and June 2023. The participants were categorized into two groups based on whether their 24-hour urinary protein levels exceeded 150 mg upon admission. Specifically, those with a 24 h urinary protein level exceeding 150 mg were classified as the IgA vasculitis nephritis (IgAVN), while the remaining participants were included in the IgAV group. A comparative assessment was performed to evaluate the general condition and laboratory examination results of both groups. The logistic regression analysis was utilized to pinpoint variables correlated with renal injury, facilitating the development of a risk prediction model. The receiver operating characteristic curve(ROC) was employed to evaluate the model's predictive performance.

**Results:**

The univariate analysis revealed that the duration of rash, gender, patient age, levels of C-reactive protein(CRP), Immunoglobulin G (IgG), albumin, globulin, glutamic oxaloacetic transaminase(AST), total cholesterol(TC), urine routine results, and 25-(OH)-D_3_ were all identified as potential influencing factors for IgAVN. Multivariate analysis revealed that albumin, patient age, and TC emerged as independent influential factors in the occurrence of IgAVN. The area under the curve (AUC) for the combined predictor(age + albumin + TC) was significantly larger than that of individual factors such as age, albumin, and TC, with respective AUC values of 0.804, 0.673, 0.737, and 0.608. The prediction model of IgAVN was further developed as follows: logit(P) = 3.978 + 0.199 × age (years) − 0.197 × albumin (g/L) + 0.550 × TC (mmol/L).

**Conclusion:**

By utilizing certain laboratory indicators, it is possible to enhance the prediction of IgAVN, thereby reinforcing the importance of follow-up measures for early detection, diagnosis, and treatment in order to mitigate the occurrence of unfavorable prognosis.

## 1 Introduction

Immunoglobulin A vasculitis (IgAV) is the most prevalent form of vasculitis. It is characterized by skin purpura in the absence of thrombocytopenia, arthralgia and arthritis, abdominal pain, and kidney damage. The disease generally follows a self-limiting course with a favorable prognosis. Among these, gastrointestinal involvement, characterized by abdominal pain, significantly influences the short-term prognosis of IgAV, while renal involvement is a critical determinant of its long-term prognosis (1, 2). Studies have shown that approximately one-third of patients with IgAV exhibit IgA vasculitis with nephritis (IgAVN). Furthermore, it is estimated that 25%–30% of individuals with IgAVN are at risk of progressing to chronic kidney disease (CKD) (3), while 1%–7% of pediatric cases of IgAVN may advance to end-stage renal disease (ESRD) (4). Consequently, the early identification of renal impairment and prompt therapeutic intervention hold substantial clinical importance for enhancing the prognosis in pediatric patients with IgAV. Previous literatures have demonstrated that the incidence of IgAVN is closely associated with the severity and duration of cutaneous purpura, as well as the presence of accompanying symptoms such as abdominal pain, gastrointestinal bleeding, and arthralgia (5). Furthermore, renal involvement typically manifests within 4–6 weeks following the diagnosis of IgAV; however, clinical identification may extend beyond 3 months or longer. In this study, we conducted a retrospective analysis of the clinical data from patients newly diagnosed with IgAV to identify early predictors of renal involvement. Additionally, we aimed to develop a risk prediction model for IgAVN to facilitate early intervention, thereby reducing the incidence and progression of IgAVN and enhancing the quality of life for affected patients.

## 2 Methods

### 2.1 Participants

Children diagnosed with IgAV admitted to our hospital from July 1, 2020 to June 30, 2023 were selected as the research objects, and the differences in clinical data were analyzed and compared. Inclusion Criteria: (1) Fulfillment of the diagnostic criteria for IgAV; (2) Initial diagnosis of IgAV; (3) The participants' ages ranged from 2 to 13 years; (4) Measurement of 24 h urinary protein (24 h-UP) prior to the administration of glucocorticoids; (5) Availability of complete clinical data. Exclusion criteria: (1) Previous diagnosis of IgAV; (2) incomplete clinical data; (3) Use of glucocorticoids, immunosuppressants, and blood products in the past 3 months; (4) Patients with underlying diseases requiring the use of glucocorticoids or immunosuppressants, such as hematological diseases, autoimmune diseases, genetic metabolic diseases, etc.

### 2.2 Clinical data

Clinical data were systematically gathered, encompassing demographic and clinical parameters such as gender, age, weight, height, body mass index (BMI), duration of the rash, white blood cell count (WBC), absolute neutrophil count (NEU), absolute lymphocyte count (LYM), neutrophil-to-lymphocyte ratio (NLR), platelet-to-lymphocyte ratio (PLR), platelet count (PLT), c-reactive protein (CRP), immunoglobulin A (IgA), immunoglobulin G (IgG), immunoglobulin M (IgM), immunoglobulin E (IgE), components C3 and C4, albumin, globulin, glutamic pyruvic transaminase(ALT), glutamic oxaloacetic transaminase(AST), total cholesterol (TC), triglyceride (TG), 25-hydroxyvitamin-D_3_(25-OH-D_3_), urine white blood cells, urine red blood cells, blood urea nitrogen (BUN), Serum Creatinine (Scr), estimated glomerular filtration rate (eGFR) and 24 h-UP. BMI = weight (kg) / height^2^ (m^2^), eGFR[ml · min^−1^ · (1.73 m^2^)^−1^] = K × height (cm)/Scr (μmol/L). The value of K was 48.6 for girls aged 2–21 years, 48.6 for boys aged 2–12 years, and 61.74 for boys aged 13–21 years. The subjects were divided into two groups according to whether the 24 h-UP was greater than 150 mg, that is, when the 24 h-UP was greater than 150 mg, it was IgAVN group.

### 2.3 Statistical analysis

The SPSS 25 software was used for statistical analysis. For univariate analysis, nonnormally distributed continuous variables were presented as *M (Q1*–*Q3)*. Normally distributed continuous variables were presented as mean ± standard deviation. Enumeration data were expressed as examples. The *Chi-square test, t-test*, and *Mann–Whitney U* test were used to perform univariate analysis on the clinical data of the participants. Binary logistic regression was selected for multivariate analysis, and a predictive model was subsequently developed. The receiver operating characteristic curve (ROC) was used to analyze the variables with *P* < 0.05 in multivariate analysis.

## 3 Results

### 3.1 Baseline characteristics of IgAV patients

**Figure 1:**
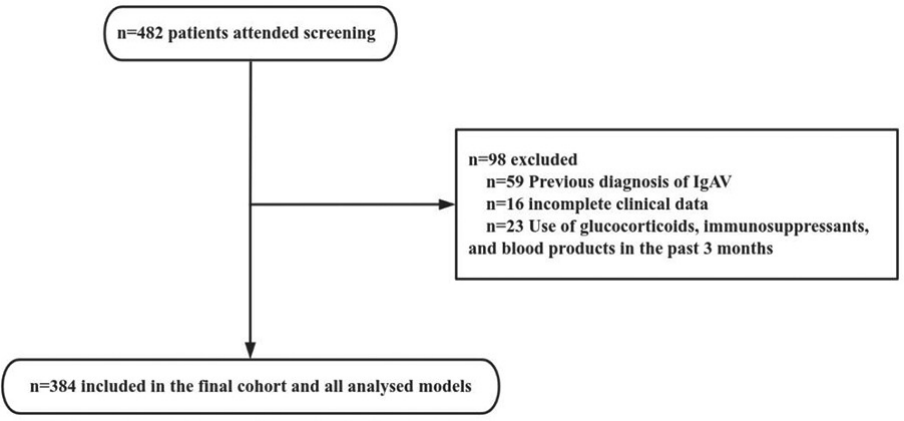
Flowchart of selection process for eligible studies.

From July 1, 2020 to June 30, 2023, a total of 482 patients diagnosed with IgAV were admitted to our hospital. After screening, 98 patients were excluded, including 59 patients with previous diagnosis of IgAV (including patients with multiple hospitalizations in our hospital or diagnosed in other hospitals), 23 patients with glucocorticoid use before diagnosis, and 16 patients with incomplete clinical data. Consequently, 384 patients were included in the study (Figure 1). The demographic, clinical, and biochemical characteristics of the 384 patients with IgAV at their initial presentation are presented in detail in Table 1.

**Table 1:** Baseline demographic, clinical and biochemical data in384 patients with IgAV.

Characteristics	Mean ± s.d.(percentage)
Male sex(n,%)	211 (54.9%)
Age(years)	7.8 ± 2.8
Body weight(kg)	32.6 ± 14.6
Body length(cm)	132.7 ± 18.4
BMI(kg/m^2^)	17.6 ± 3.7
Duration of rash(days)	9.3 ± 11.6
WBC(10^9^/L)	10.7 ± 4.2
NEU(10^9^/L)	7.0 ± 3.9
LYM(10^9^/L)	2.9 ± 1.3
NLR	3.1 ± 2.6
PLR	140.9 ± 78.9
PLT(10^9^/L)	336.6 ± 82.6
CRP(mg/L)	6.7 ± 15.3
IgA(g/L)	2.3 ± 1.0
IgG(g/L)	10.6 ± 2.8
IgM(g/L)	1.3 ± 0.5
IgE(IU/ml)	195.4 ± 432.5
C3(g/L)	1.0 ± 0.2
C4(g/L)	0.2 ± 0.1
Albumin(g/L)	42.1 ± 4.2
Globulin(g/L)	33.5 ± 94.3
ALT(U/L)	16.2 ± 20.0
AST(U/L)	26.5 ± 10.8
TC(mmol/L)	4.4 ± 1.0
TG(mmol/L)	1.2 ± 0.9
25-OH-D3(ng/ml)	24.5 ± 63.7
BUN(mmol/L)	4.5 ± 1.2
Scr(umol/L)	33.3 ± 8.1
eGFR[ml·min^−1^·(1.73m^2^)^−1^]	151.2 ± 28.9
Urinary leukocytes(cells/ul)	9.2 ± 37.2
Urine erythrocyte(cells/ul)	24.2 ± 171.4

CRP, C-reactive protein; IgG, immunoglobulin G; AST, glutamic oxaloacetic transaminase; TC, total cholesterol; WBC, white blood cell count; NEU, neutrophil count; LYM, lymphocyte count; NLR, neutrophil-to-lymphocyte ratio; PLR platelet-to-lymphocyte ratio; PLT, platelet count; IgA, immunoglobulin A; IgM, immunoglobulin M; IgE, immunoglobulin E; ALT, glutamic pyruvic transaminase; TG, triglyceride; 25-OH-D_3_, 25-hydroxyvitamin-D_3_; BMI, body mass index; BUN, blood urea nitrogen; Scr, serum creatinine; eGFR, estimated glomerular filtration rate.

### 3.2 Univariable analyses

**Figure 2:**
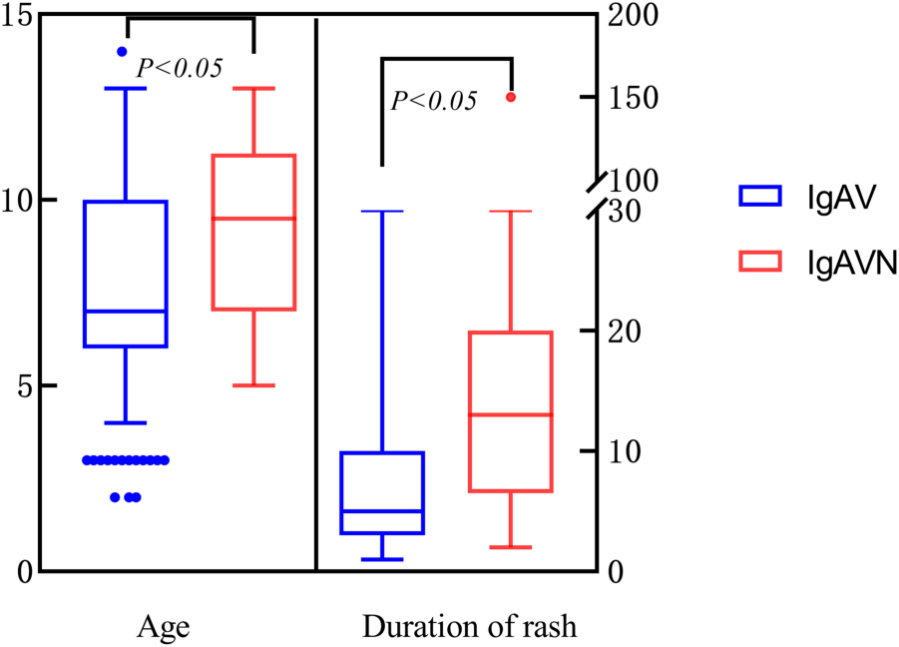
Univariate analysis of Age and duration of rash.

This study encompassed a total of 384 cases of IgAV, of which 54 cases were specifically diagnosed with IgAVN. Upon comparing the demographic characteristics of the two groups, it was observed that the male predominance was consistent across both cohorts. Additionally, the IgAVN group exhibited a significantly higher mean age and longer duration of rash, with statistically significant differences noted (Table 2; Figure 2). In terms of hematological parameters, only CRP levels showed a statistically significant difference between the two groups (Table 2). The comparison of immune function revealed a statistically significant difference in IgG levels between the two groups (Table 2). Furthermore, this study identified statistically significant differences in albumin, globulin, AST, TC, 25-OH-D_3_, and urinalysis results between the two groups (*P* < *0.05*) (Table 2).

**Table 2:** Univariable analyses.

Characteristics	No renal injury(330)	IgAVN(54)	χ^2^/Z	*P*
Male	179 (54.2)	32 (59.3)	0.472	0.492
Age(years)	7 (6,10)	10 (7,12)	−4.095	<0.001
BMI(kg/m^2^)	16.6 (15.0,19.5)	17.1 (15.2,19.5)	−0.988	0.323
Duration of rash(days)	5.0 (3.0,10.0)	13.0 (6.5,20)	−5.621	<0.001
WBC(10^9^/L)	9.6 (7.9,12.1)	10.0 (7.2,13.9)	−0.022	0.983
NEU(10^9^/L)	5.6 (4.4,7.9)	7.1 (4.3,8.7)	−0.435	0.663
LYM(10^9^/L)	2.7 (1.9,3.6)	2.4 (1.7,3.2)	−0.781	0.435
NLR	2.1 (1.4,3.7)	2.5 (1.4,4.8)	−0.681	0.496
PLR	119.4 (88.8,164.9)	125.7 (86.2,168.5)	−0.499	0.618
PLT(10^9^/L)	331.5 (274.0,384.3)	296.0 (252.0,359.0)	−0.729	0.466
CRP(mg/L)	1.8 (0.5,6.3)	0.9 (0.2,5.1)	−2.136	0.033
IgA(g/L)	2.1 (1.6,2.9)	2.6 (2.1,3.2)	−1.641	0.101
IgG(g/L)	10.6 (9.1,12)	10.3 (8.3,12.9)	−2.985	0.003
IgM(g/L)	1.2 (0.9,1.6)	1.2 (0.9,1.8)	−0.109	0.913
IgE(IU/ml)	67.5 (25.5,152.5)	53.6 (26.2,116.0)	−0.981	0.326
C3(g/L)	1.0 (0.9,1.1)	0.9 (0.8,1.0)	−1.076	0.282
C4(g/L)	0.2 (0.2,0.3)	0.2 (0.2,0.2)	−0.891	0.373
Albumin(g/L)	43.0 (40.4,44.8)	40.8 (37.8,42.4)	−4.121	<0.001
Globulin(g/L)	27.1 (24.9,29.1)	26.6 (24.1,29.7)	−2.464	0.014
ALT(U/L)	12.0 (9.0,15.0)	12.0 (10.0,15.0)	−1.821	0.069
AST(U/L)	25.0 (21.0,29.0)	20.0 (18.0,28.0)	−1.956	0.049
TC(mmol/L)	4.1 (3.6,4.9)	4.4 (4.1,5.6)	−2.425	0.015
TG(mmol/L)	1.0 (0.7,1.4)	0.8 (0.7,1.3)	−0.658	0.511
25-OH-D3(ng/ml)	20.8 (14.6,26.3)	14.5 (10.4,21.8)	−3.480	0.001
BUN(mmol/L)	4.4 (3.6,5.1)	4.3 (4.0,5.6)	−1.385	0.166
Scr(umol/L)	32.8 (26.9,38.8)	33.7 (30.0,37.5)	−1.273	0.203
eGFR[ml·min^−1^·(1.73m^2^)^−1^]	147.3 (130.1,166.5)	151.4 (136.4,173.7)	−1.621	0.105
Urinary leukocytes(cells/ul)	1.0 (0.0,4.0)	3.0 (0.0,6.0)	−5.018	<0.001
Urine erythrocyte(cells/ul)	0.0(0.0,0.0)	1.0(0.0,20.0)	−8.357	<0.001

IgAVN, immunoglobulin A vasculitis with nephritis; CRP, C-reactive protein; IgG, immunoglobulin G; AST, glutamic oxaloacetic transaminase; TC, total cholesterol; WBC, white blood cell count; NEU, neutrophil count; LYM, lymphocyte count; NLR, neutrophil-to-lymphocyte ratio; PLR, platelet-to-lymphocyte ratio; PLT, platelet count; IgA, immunoglobulin A; IgM, immunoglobulin M; IgE, immunoglobulin E; ALT, glutamic pyruvic transaminase; TG, triglyceride; 25-OH-D_3_, 25-hydroxyvitamin-D_3_; BMI, body mass index; BUN, blood urea nitrogen; Scr, serum creatinine; eGFR, estimated glomerular filtration rate.

### 3.3 Multivariate analysis

Binary logistic regression model was used to further analyze the variables with single factor *P* < *0.05*. The results indicated that the duration of rash, CRP levels, IgG, globulin, AST, 25-OH-D_3_, and urine routine parameters were not independently associated IgAVN (*P* > 0.05) (Table 3). In contrast, age, albumin, and TC were independent predictors of IgAVN(*P* < 0.05), with advanced age, reduced serum albumin levels, and elevated TC being significant risk factors for the development of IgAVN (Table 3).

**Table 3:** Multivariate analysis.

Factors	β	S.E.	Wald*χ*^2^	Exp	*95%CI*	*P*
Age	0.199	0.100	3.934	1.220	1.002–1.486	0.047
Duration of rash	0.028	0.015	3.602	0.058	0.999–1.059	0.058
CRP	0.007	0.016	0.203	1.007	0.977–1.039	0.652
IgG	0.018	0.153	0.015	1.019	0.755–1.373	0.904
Albumin	−0.197	0.069	8.169	0.821	0.718–0.940	0.004
Globulin	−0.037	0.114	0.106	0.963	0.770–1.206	0.745
AST	−0.030	0.025	1.443	0.971	0.925–1.019	0.230
TC	0.550	0.214	6.620	1.733	1.140–2.633	0.010
25-OH-D_3_	−0.043	0.033	1.698	0.958	0.899–1.022	0.193
Urinary leukocytes	0.006	0.009	0.433	1.006	0.989–1.023	0.511
Urine erythrocyte	0.004	0.003	1.694	1.004	0.998–1.009	0.193
Constant	3.978	3.097	1.649	53.389		0.199

### 3.4 The ROC curves for various variables predicting IgAVN

**Figure 3:**
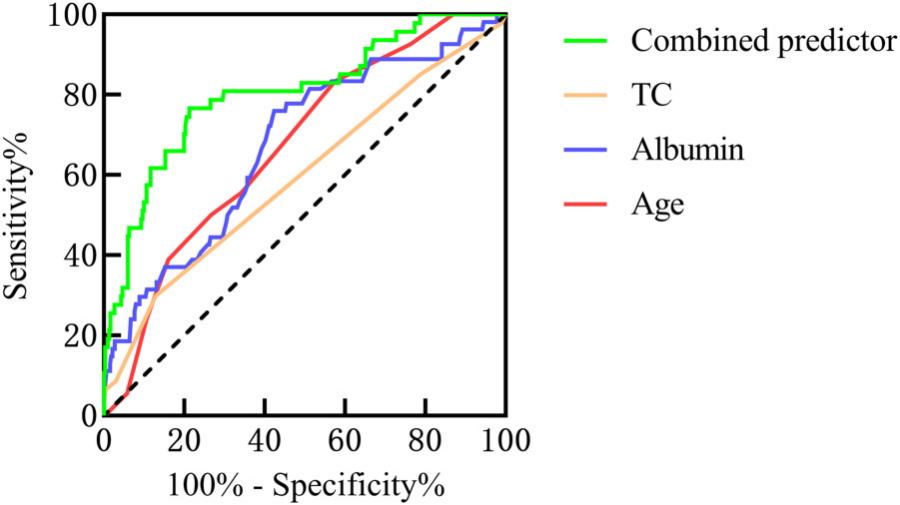
The ROC curves for various variables predicting IgAVN.

The AUC for the combined predictors(age + albumin + TC) was significantly higher than that of age, albumin, or TC alone in predicting the occurrence of IgAVN (Figure 3).

### 3.5 Construction of the prediction model

The prediction model for IgAVN based on binary logistic regression analysis was further developed as follows (Table 2): logit(P) = 3.978 + 0.199 × age (years) − 0.197 × albumin (g/L) + 0.550 × TC (mmol/L). The model fit was tested by Hosmer-Lemeshow test, *χ^2^* = 12.917, *P* = 0.115 > 0.05, indicating that the regression equation had statistical significance.

## 4 Discussion

IgAVN can occur at any age, and the peak age of onset and incidence are different in different countries and regions. Approximately 90% of these cases are observed in children aged 2–10 years (6), with a peak incidence occurring between the ages of 4 and 7 years (7). Additionally, the prevalence of IgAV is marginally higher in boys compared to girls (7). The age range of IgAV patients in this study was from 2 to 13 years. Among these, 49.2% of cases were observed in the 4–7 year age group, and 54.9% were male patients. These findings are consistent with previous literature. Concurrently, the incidence of IgAVN among the 384 patients was 14.1%. Previous studies have demonstrated that approximately one-third of IgAV patients exhibit characteristics of IgAVN (8). The lower incidence of IgAVN in this study can be attributed to two primary factors: (1) Variations in the definition of IgAVN: In this study, IgAVN was defined as proteinuria exceeding 150 mg over 24 h. The clinical manifestations of IgAVN were diverse, encompassing isolated hematuria, isolated proteinuria, hematuria combined with proteinuria, acute nephritis, nephrotic syndrome, rapidly progressive glomerulonephritis, and chronic nephritis. In the study, no patients of gross hematuria were observed, and only a limited number of patients exhibited microscopic hematuria. The median count of red blood cells in the urine routine analysis was 0 for the non-renal injury group, while it was 1 for the IgAVN group. Additionally, all patients with microscopic hematuria demonstrated varying degrees of proteinuria. Consequently, in this study, the criterion for defining IgAVN was based solely on 24-hour urinary protein levels (>150 mg), without separate analysis of hematuria. Additionally, given that this study is a retrospective analysis and inherently possesses certain limitations, dynamic follow-up of these patients will be conducted in the future to facilitate more in-depth and comprehensive research. (2) Differences in the timing of IgAVN diagnosis: In this study, the onset time of IgAVN was defined as the initial diagnosis time of IgAV patients, with a median duration of skin purpura of 10 days. According to relevant literature, 97% of pediatric cases exhibited renal involvement within six months of disease onset, and 85% developed renal damage within one month (9). Additionally, The relevant studies in China (10) reported that 96.7% of children developed renal damage within 6 months, with 73.40% manifesting within 1 month.

The findings of this study revealed that the median age of onset for the IgAVN group was 10 years (interquartile range: 7–12 years). Multivariate logistic regression analysis indicated that age is an independent risk factor for the development of IgAVN, suggesting a positive correlation between increasing age and the incidence of IgAVN. Xu H et al. (11) analyzed data from 669 patients with IgAV and found that the mean onset age of the IgAVN group was significantly higher than that of the non-renal injury group (8.9 ± 2.7 years vs. 6.8 ± 2.6 years), which consistent with the conclusion of this study. However, the specific age at which IgAVN is more likely to occur is inconsistent among different studies, such as Demircioğlu et al. (5) showing that age greater than 8 is a risk factor for IgAVN, while Zheng et al. (12) showed that age greater than 6 years is a risk factor for IgAVN. The discrepancies in these results can be attributed to subtle differences in patient grouping criteria across studies. Furthermore, owing to the limited sample size of IgAVN in this study, a detailed analysis of the age of IgAVN onset was not conducted. However, regardless of the grouping method employed, the consistent conclusion was drawn that the risk of IgAVN increases with advancing age.

Serum albumin is the most abundant protein in plasma, playing a vital role in sustaining the body's nutritional status and colloidal osmotic pressure. In the presence of kidney injury or disease, particularly when the glomerular filtration membrane is compromised, a significant loss of proteins occurs, leading to a reduction in serum albumin levels. Researches had demonstrated that hypoproteinemia was closely associated with impaired renal function and the progression and prognosis of nephropathy (13, 14). The study conducted by Coppo et al. (15) demonstrated that serum albumin levels played a crucial role in assessing the condition and prognosis of IgAVN, findings which were consistent with those reported by Mao et al. (16). In our study, serum albumin levels were significantly lower in the IgAVN group compared to the non-renal injury group (40.8 vs. 43, *p* < 0.05), with a statistically significant difference. Additionally, serum albumin was an independent protective factor against the development of IgAVN (*P* = 0.004, OR = 0.821, *95% CI*: 0.718–0.940). Consequently, clinicians should be vigilant regarding the potential onset of IgAVN in patients with IgAV who exhibit low serum albumin levels.

It is widely recognized that lipid metabolism disorders serve as significant risk factors for the onset and progression of cardiovascular diseases, including atherosclerosis, coronary heart disease, and hypertension (17). In the context of kidney disease, dyslipidemia not only represents a frequent clinical manifestation of numerous primary renal conditions but is also intricately associated with the advancement and prognosis of renal disease (18, 19). In recent years, numerous studies have demonstrated a significant association between dyslipidemia and the progression and prognosis of Immunoglobulin A nephritis(IgAN) (13, 20). Specifically, hypertriglyceridemia and hypercholesterolemia have been shown to elevate the risk of hypertension and proteinuria in patients with IgAN, which are critical risk factors for disease progression (21, 22). Wang et al. (23) reported that IgAN patients with hypertriglyceridemia exhibited a markedly reduced renal survival rate, indicating that hypertriglyceridemia is an independent risk factor for poor prognosis in these patients. However, most lipid-related studies have primarily focused on adult IgAN populations, with limited research available on pediatric cases and even fewer studies on IgAVN. Meanwhile, lipids are a very broad concept, encompassing fats, phospholipids, and steroids. This study utilized TC as a model to investigate its role in the development of IgAVN in pediatric patients. The findings revealed that TC levels in the IgAVN group were significantly higher compared to those in the non-kidney injury group (4.4 vs. 4.1), with a statistically significant difference between the two groups. Furthermore, TC was an independent risk factor for the occurrence of IgAVN (*P* = 0.010; *OR* = 1.733, *95% CI* 1.140–2.633). The study by Wu et al. (24) on TC levels in healthy children, children with IgAVN, and IgAV patients without renal injury demonstrated that TC levels were significantly elevated in both IgAVN and IgAV patients without renal injury compared to healthy controls. Therefore, abnormal lipid metabolism in the early stage of IgAV can predict the risk of renal damage to a certain extent, effectively guide the early detection and treatment of children, and block the progression of the disease. However, given the limited number of studies, the relatively small sample size, and the single-center nature of this research, the findings may be subject to certain limitations.

Previous literatures (25, 26) have demonstrated that patients with persistent or recurrent cutaneous purpura are at a higher risk of developing IgAVN. In this study, a significant difference was observed in the duration of skin purpura between the two groups. Specifically, the IgAVN group exhibited a longer duration of skin purpura compared to the non-renal injury group (13 vs. 5). However, multivariate logistic regression analysis indicated that the duration of skin purpura was not an independent risk factor for IgAVN (*P* = 0.058, *OR* = 1.039, *95% CI*: 0.999–1.059). Since the duration of cutaneous purpura was recorded only at the initial visit in this study, long-term follow-up is essential to elucidate its potential role in IgAVN. Additionally, the comparison of CRP levels between the IgAVN group and the non-renal injury group revealed that CRP levels were significantly lower in the IgAVN group compared to the non-renal injury group (0.94 *vs*. 1.8), with a statistically significant difference (*P* < 0.05). However, CRP cannot be considered an independent predictor for the development of IgAVN, as indicated by the lack of statistical significance (*P* = 0.652, OR = 1.007, 95% CI: 0.977–1.039). Currently, there is limited literature on CRP in the context of IgAVN, and further research is warranted to clarify its clinical significance. At the same time, with regard to common risk factors for IgAV nephropathy such as renal function , eGFR and other variables, it was observed that there were statistically significant differences between the two groups in terms of BMI, BUN, Scr, and eGFR (*P* > 0.05). This conclusion may be attributable to the specific characteristics of the study population. The study focused on patients with newly diagnosed IgAV. The median onset time for the IgAVN group was 13 days, while that for the non-renal injury group was 5 days. Given the relatively short disease duration, renal function parameters (urea nitrogen, creatinine) and eGFR had not yet exhibited abnormalities. Additionally, this investigation represents a single-center, retrospective analysis with a limited sample size, necessitating further research for comprehensive insights.

Our study acknowledges several limitations. Firstly, the sample size of IgAVN cases was relatively limited and derived from a single center, which may affect the generalizability of our findings. Secondly, this study is cross-sectional in nature and does not provide prospective analysis on the long-term prognosis of IgAVN patients with respect to factors such as age, albumin levels, and cholesterol levels. In this study, 384 patients with IgAV were enrolled, of whom 54 presented with IgAVN at their initial visit. Renal biopsies were performed in 27 cases, revealing ISKDC grade IIIb in 3 cases, IIIa in 14 cases, IIb in 1 case, and IIa in 9 cases. Only one patient underwent renal biopsy at the initial visit, which was classified as ISKDC grade IIa. Due to the limited number of renal biopsies conducted, the correlation between initial diagnostic findings and subsequent renal pathology could not be further explored. To further enhance research in this area, future studies should consider increasing the sample size and expanding the number of study centers to improve the external validity of the findings. Additionally, adopting a longitudinal study design would facilitate a more comprehensive understanding of the long-term prognosis of IgAVN patients and its associated influencing factors. Through regular follow-ups with patients, researchers can better document changes in their condition across different stages, thereby providing a more robust foundation for clinical decision-making.

## Data Availability

The raw data supporting the conclusions of this article will be made available by the authors, without undue reservation.
